# Loss of fungal sensing exacerbates liver injury in a murine model of MASLD

**DOI:** 10.1172/jci.insight.190690

**Published:** 2026-03-10

**Authors:** Vijay Pandyarajan, So Yeon Kim, Takashi Tsuchiya, Selena Liu, Sadam H. Bhat, Jieun Kim, David M. Underhill, Mazen Noureddin, Shelly C. Lu, Ekihiro Seki

**Affiliations:** 1 Karsh Division of Gastroenterology and Hepatology, Department of Medicine, and; 2 Department of Biomedical Sciences, Cedars-Sinai Medical Center, Los Angeles, California, USA.; 3 Houston Methodist Hospital, Houston, Texas, USA.; 4 Houston Research Institute, Houston, Texas, USA.

**Keywords:** Gastroenterology, Hepatology, Hepatitis, Obesity

## Abstract

Metabolic dysfunction–associated steatotic liver disease (MASLD) is a global health concern with limited interventions. While the role of gut bacteria in MASLD has been extensively studied, the contribution of gut fungi remains largely unexplored. This study investigates the impact of fungal dysbiosis and the role of CARD9, a key adaptor protein in fungal sensing on gut-liver axis dysfunction in MASLD. Patients with advanced liver fibrosis exhibited distinct mycobiota profiles. Using a *Card9*-deficient mouse model subjected to high-fat, high-glucose/-fructose feeding, we observed exacerbated liver injury and fibrosis accompanied by fungal dysbiosis, paralleling our findings in human patients. Beyond its established expression in myeloid cells, *CARD9* was also detected in intestinal enterocytes where its expression was diminished under metabolic stress. Intestinal organoids with CARD9 inhibition had reduced expression of antimicrobial Reg3g, the tight junction protein ZO-1, and the antifungal enteroendocrine hormone PYY. These findings suggest that CARD9 maintains gut barrier integrity, preventing microbial translocation and subsequent liver injury and fibrosis. Our results provide insights into the interplay between fungal dysbiosis, gut barrier dysfunction, and MASLD, and identify CARD9 as a key protein within this axis.

## Abstract 2

The fungal dysbiosis and dysfunction of CARD9, an adaptor protein in fungal sensing, play key roles in the development of metabolic dysfunction-associated steatotic liver disease.

## Introduction

Metabolic dysfunction–associated steatotic liver disease (MASLD), formerly known as non-alcoholic fatty liver disease (NAFLD), is a disease characterized by excessive lipid accumulation in the liver. It is currently estimated to affect nearly 40% of the world’s population and its prevalence is continuing to escalate on this global scale (1, 2). This pandemic has increasingly led to rising health care costs and burden on the medical system. Based on Markov models, it is estimated that the cumulative cost for treating these patients will rise to approximately $1.7 trillion, resulting in the health care cost per patient increasing from $3,536 to $6,968 (3). Non-economic or indirect costs are also continuing to rise significantly and quality-of-life measures are increasingly also impacted by this condition (4). With limited approved medical pharmaceutical therapies for this condition, it is becoming increasingly apparent that this is a problem that needs solutions sooner rather than later.

MASLD encompasses a broad range of disease phenotypes. Specifically, steatotic liver disease is present when there is greater than 5% lipid accumulation in the liver (5). The manifestation of this can range from simple steatosis to metabolic dysfunction–associated steatohepatitis (MASH) to cirrhosis and even the development of hepatocellular carcinoma. Approximately 25% of patients with MASLD traverse through this spectrum to more advanced fibrotic states (6). The pathogenic underpinnings of MASLD encompass a multitude of factors that contribute to the diverse spectrum of clinical phenotypes observed. The gut microbiome is now emerging as a risk factor in various diseases (7). This complex ecosystem comprises a diverse array of organisms, including, but not limited to, bacteria, archaea, fungi, and viruses. Research has increasingly highlighted the pivotal role of these microorganisms in conditions traditionally not associated with the gut, such as autism and heart disease (8, 9). Functioning as an extra-corporeal “organ,” the gut microbiome has been shown to have profound impacts on energy absorption, nutrient utilization, and adiposity regulation. Illustrating this concept are seminal experiments involving fecal transplantation from obese mice to conventional mice, resulting in weight gain and increased hepatic steatosis (10). This has led to an emerging concept that obesity can be transmitted via the microbiome. More importantly though, this also suggests that changes within the microbiome could theoretically lead to amelioration or improvement of these disease phenotypes (11).

Fungi, a frequently overlooked component of the gut microbiome, have remained largely unexplored in the literature (12). This is largely attributed to the dominance of bacteria in the gut and the technical challenges posed by fungal cell wall lysis during DNA extraction protocols. A role for fungi in the development of liver disease has already been established (13). For instance, the mycotoxin aflatoxin, is widely recognized for its role in the development of hepatocellular carcinoma (14). Moreover, fungal involvement in alcoholic liver disease has also been substantiated (15). Existing investigations into patients with MASLD have predominantly focused on ethnically homogeneous populations, which may limit their generalizability to the diversity observed in the United States (16, 17). These prior investigations have determined that overgrowth of potentially pathogenic fungi such as *Candida* may play a central role in contributing to disease progression. This suggests that, in the disease state, there is a form of relative fungal immunodeficiency.

To investigate the role of fungal immunity further, we identified potential murine models that could illuminate the interaction between antifungal immunity and MASLD progression. Among key proteins involved in microbial recognition and inflammation, we focused on MyD88, NLRP3, and caspase recruitment domain protein 9 (CARD9). While MyD88 and NLRP3 play broad roles in bacteria-, virus-, and damage-associated inflammatory responses, CARD9’s specificity in fungal recognition made it a compelling candidate (18–20). Based on its central role in fungal immunity and gut microbial homeostasis, we selected a murine model of CARD9 deficiency to further investigate the impact of fungal dysbiosis and antifungal immunity in MASLD.

CARD9 is a critical signaling protein involved in the immune response to fungal pathogens, particularly through recognition of fungal cell wall components (20). Predominantly expressed in myeloid cells, CARD9 is essential for gut immunity, and disruptions in its signaling are associated with gut dysbiosis, exemplified by the reduction of beneficial bacteria such as *Lactobacillus reuteri* and expansion of pathogenic fungi related to Crohn disease such as the genus *Debaryomyces* in *Card9*-deficient (*Card9^–/–^*) mice (21). While CARD9 has been linked to intestinal inflammation, its role in MASLD remains relatively unexplored. Using a global CARD9–knockout model in conjunction with a high-fat, high-sugar feeding model of diet-induced obesity, we demonstrate that *Card9^–/–^* mice exhibited increased liver injury and fibrosis and displayed microbial dysbiosis. These findings suggest that impaired fungal sensing may contribute to the progression of MASLD. Intriguingly, in both our animal model and human cohorts, our results also show that a loss of the *Penicillium* genus may be correlated with advanced liver disease. This suggests that *Penicillium* may be a beneficial fungus that prevents or ameliorates disease progression.

## Results

### The microbiota profiles of those with mild and severe liver fibrosis are distinct in a diverse population of patients with MASLD.

We first began our investigation by determining whether the intestinal microbiome is associated with MASLD development. We performed targeted next-generation sequencing for both bacterial and fungi DNA via amplicon sequencing of the *16S* region of bacterial DNA and the internally transcribed spacer region 1 (*ITS1*) region of the rRNA region of fungi, respectively. We obtained 17,322 average reads per sample (range 1471–119,264). We analyzed a total of 62 patients. These patients were stratified based on hepatic fibrosis status into mild (F0–F1, *n* = 34) and severe categories (F2–F4, *n* = 28) (Table 1). Fibrosis status was determined either through liver biopsy or through non-invasive studies (transient elastography or magnetic resonance elastography). There was a significant difference in the fibrosis determination method, as assessed by χ^2^ tests showing a slight increase in biopsies in the more advanced liver fibrosis group. The average age of our cohort was 57.1 ± 12.1 years and 60.6 ± 8.6 years for the mild and severe fibrosis categories, respectively. There was a male predominance in both categories (61.8% and 53.6%). The average BMI for each category (mild, 32.7 ± 5.7 kg/m^2^ and severe, 31.2 ± 6.8 kg/m^2^) placed each fibrosis group into the obese designation. In contrast to prior studies where the fecal mycobiome had been described in patients with MASLD, our cohort consisted of a diverse ethnic population that included a large proportion of individuals of Hispanic origin (mild, 35.3% and severe 21.4%), a population with elevated rates of fibrosis progression (22). Aspartate aminotransferase (AST) and platelet counts were found to be significantly increased and decreased respectively in the F2–F4 fibrosis group.

**Figure 1:**
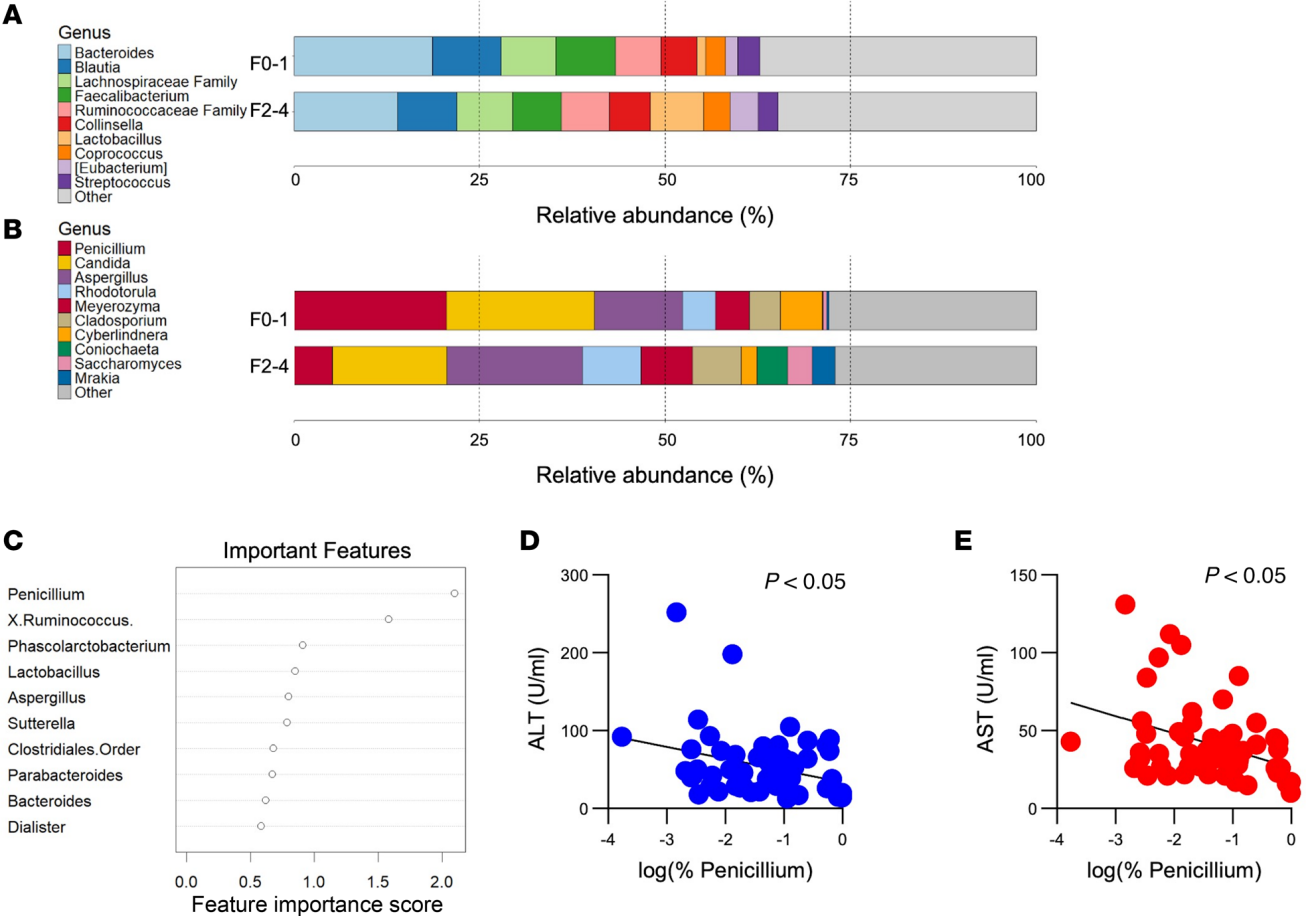
Microbiota changes in a cohort of patients with MASLD stratified by F0–F1 (*n* = 34) and F2–F4 (*n* = 28) fibrosis stages. (**A**) Bacterial *16S* sequencing (top) and (**B**) fungal *ITS1* sequencing (bottom) of stool. (**C**) Mean Gini decrease in important features identified in combined bacterial/fungal sequencing data. (**D**) Correlation of log of relative abundance with ALT and (**E**) AST.

At the bacterial level, taxonomic analysis revealed that the top 10 represented genera included *Bacteroides*, *Blautia*, *Lachnospiraceae* family, *Faecalibacterium*, *Ruminococcaceae* family, *Colinsella*, *Lactobacillus*, *Coprococcus*, *Eubacterium*, and *Streptococcus*. Genera outside of these were grouped into an “other” category (Figure 1A). We observed an expansion of *Lactobacillus* and *Eubacterium* and a reduction in *Bacteroides* and *Faecalibacterium* when we compared the severe and mild categories. In contrast to bacteria where changes in the microbiota had only slight changes, fungal mycobiota changes were greater in magnitude. The top 10 fungal mycobiota by relative abundance across both disease categories were *Penicillium*, *Candida*, *Aspergillus*, *Rhodotorula*, *Meyerozyma*, *Cladosporium*, *Cyberlindnera*, *Coniochaeta*, *Saccharomyces*, and *Mrakia*. Of these, *Penicillium* displayed the most dramatic reduction in abundance in the severe category (Figure 1B). Genera that were expanded in the severe category included *Aspergillus*, *Rhodotorula*, *Meyerozyma*, *Cladosporium*, *Sacchromyces*, and *Mrakia*. *Coniochaeta* was found to only be present in large quantities in the more severe category. No differences in α diversity were detected between the 2 groups but there was a significant difference in β diversity for the fungal data when comparing the 2 groups (Supplemental Figures 1 and 2; supplemental material available online with this article; https://doi.org/10.1172/jci.insight.190690DS1).

To determine which features, bacterial or fungal, may be important for discriminating between the mild and severe groups, we performed random forest analysis. When we combined both our bacterial and fungal data into a single data set for analysis, we interestingly found that *Penicillium* led to the greatest mean decrease in Gini accuracy, indicating it was the most important discriminative factor to distinguish between mild and severe groups (Figure 1C). Furthermore, we found significant negative correlations between aminotransferases and the abundance of *Penicillium* (Figure 1, D and E). Together, these results indicate that *Penicillium* is potentially a beneficial fungus given its decreased abundance in the severe fibrotic group.

### Amphotericin B treatment ameliorates liver injury and fibrosis under metabolic stress conditions.

**Figure 2:**
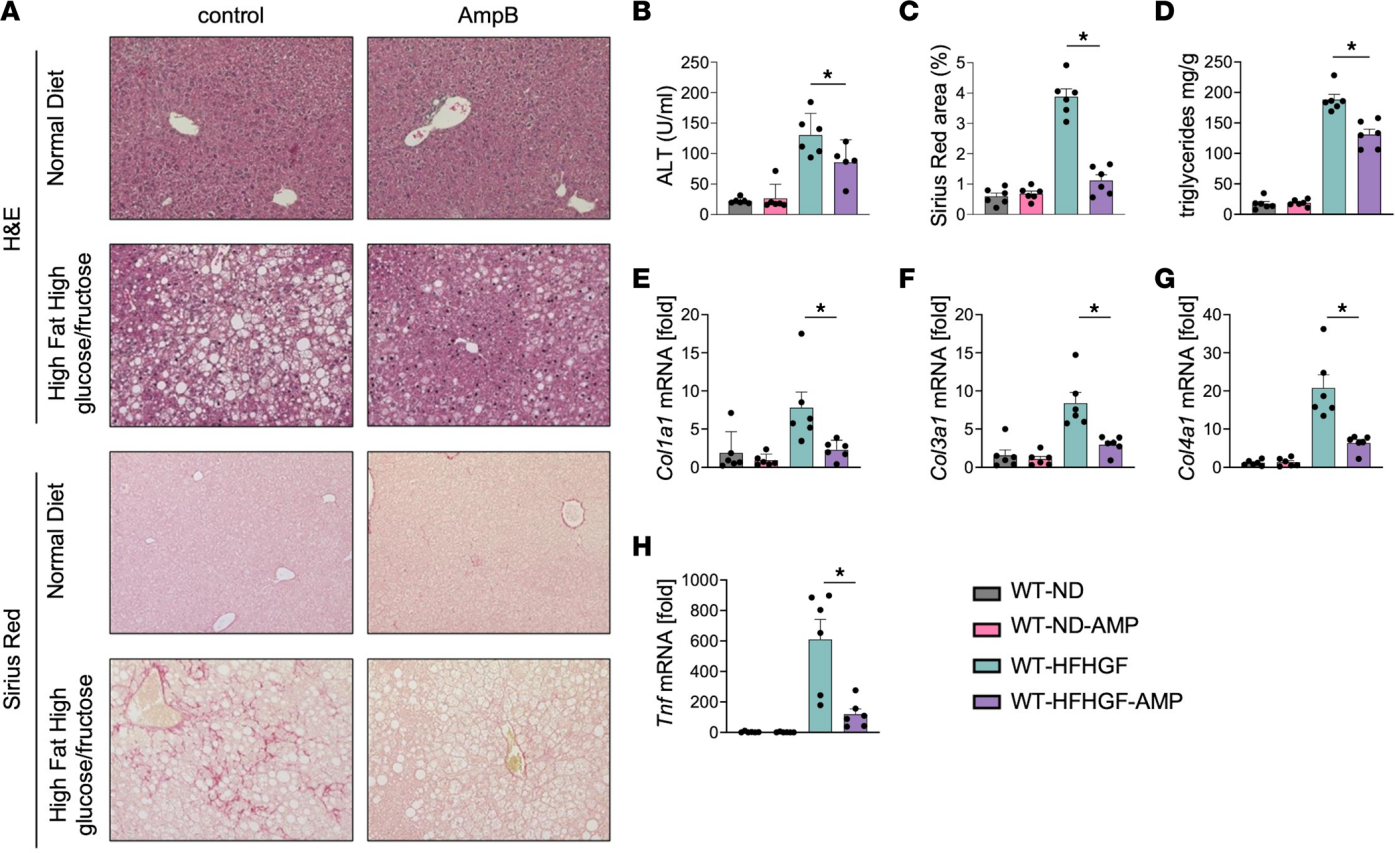
Amphotericin B ameliorates MASLD. (**A**) H&E and Sirius red staining of liver sections from WT mice fed normal diet (WT-ND, *n* = 6), WT-ND mice with amphotericin B (WT-ND-AMP, *n* = 6), WT mice fed high-fat, high-glucose/-fructose diet (WT-HFHGF, *n* = 6), and WT-HFHGF-AMP mice (*n* = 6). Original magnification, ×20. (**B**) Serum ALT. (**C**) Sirius red quantitation. (**D**) Hepatic triglyceride content. (**E**–**F**) mRNA expression for (**E**) *Col1a1*, (**F**) *Col3a1*, (**G**) *Col4a1*, and (**H**) *Tnf*. Statistical significance was calculated with 1-way ANOVA followed by Holm-Šídák multiple-comparison test (**B**–**H**). *P < 0.05.

Given the changes in fungal mycobiota in our patients, we posited that changes in the mycobiota might be associated with the development of MASLD. We therefore used a murine diet-induced model of high-fat, high-glucose/-fructose (HFHGF) feeding to induce hepatic steatosis and fibrosis in wild-type (WT) mice and coupled this to amphotericin B treatment, an antifungal compound with poor bioavailability and compared this to animals on a normal diet (ND). We found that amphotericin B–treated mice on an HFHGF diet have improved hepatic injury markers, steatosis and fibrosis, suggesting a functional contribution of gut fungi in the development of MASLD (Figure 2, A–D) and further confirming prior published results (16). At the mRNA level, there was a concomitant reduction in fibrosis markers, including *Col1a1*, *Col3a1*, and *Col4a1* and a marked reduction in *Tnf* expression in the liver (Figure 2, E–H). We then sought to define the changes in the gut microbiota in these animals using next-generation sequencing given the antifungal role of amphotericin B.

**Figure 3:**
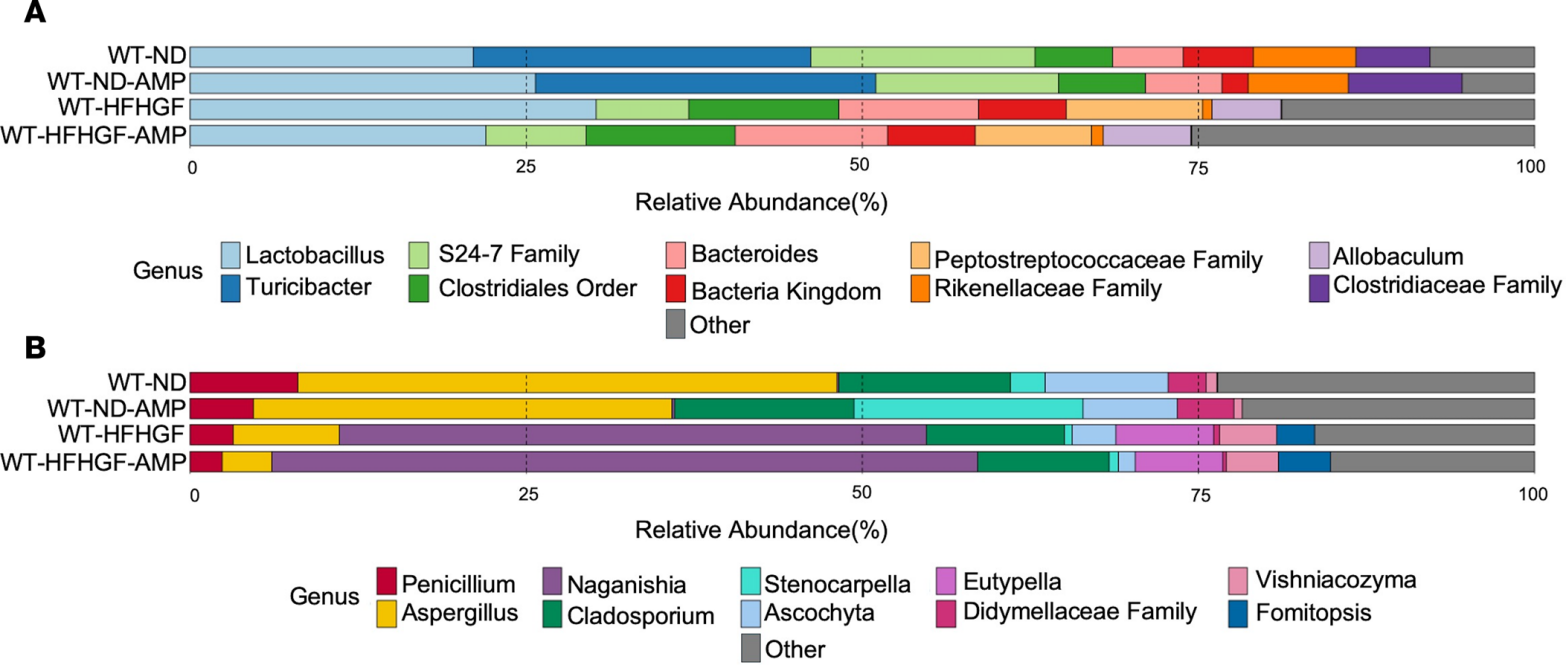
Treatment with amphotericin B (AMP) alters gut microbiota. WT mice fed normal diet (WT-ND, *n* = 6), WT-ND mice with AMP (WT-ND-AMP, *n* = 6), WT mice fed high-fat, high-glucose/-fructose diet (WT-HFHGF, *n* = 6), and WT-HFHGF mice with AMP (WT-HFHGF-AMP, *n* = 6). (**A**) Top 10 bacterial genera by relative abundance determined by *16S* sequencing of stool and (**B**) top 20 fungal genera determined by *ITS1* sequencing. Data represented as average relative abundance.

At the bacterial level, the greatest changes were between the ND and HFHGF groups. This includes the complete loss of *Turicibacter* and *Clostridiaceae* family, a reduction in the *S24-7* family, growth of the *Clostridiales* order, *Bacteroides*, and *Akkermansia* and the appearance of *Peptostreptococcaceae* family and *Allobaculum* (Figure 3A). Only subtle differences existed between the amphotericin B–treated and non-treated groups were apparent within our cohort. Like bacteria, the greatest differences were apparent between the ND and HFHGF groups. These changes include a marked reduction in *Aspergillus* and *Ascochyta* and expansion of *Eutypella*, *Vishniacozyma*, and *Fomitopsis* genera (Figure 3B). When examined more closely, amphotericin B treatment appeared to reduce *Aspergillus* in both the ND and HFHGF conditions with an expansion of the *Stenocarpella* genus in the ND condition. Notably, as described in our human cohort, animals under metabolic stress appeared to have a reduction in the *Penicillium* genus, with a slight reduction in abundance in the amphotericin B–treated groups. These results suggest that amphotericin B treatment reduces MASLD-promoting fungi.

### CARD9 deficiency leads to worse liver injury and fibrosis under metabolic stress conditions.

**Figure 4:**
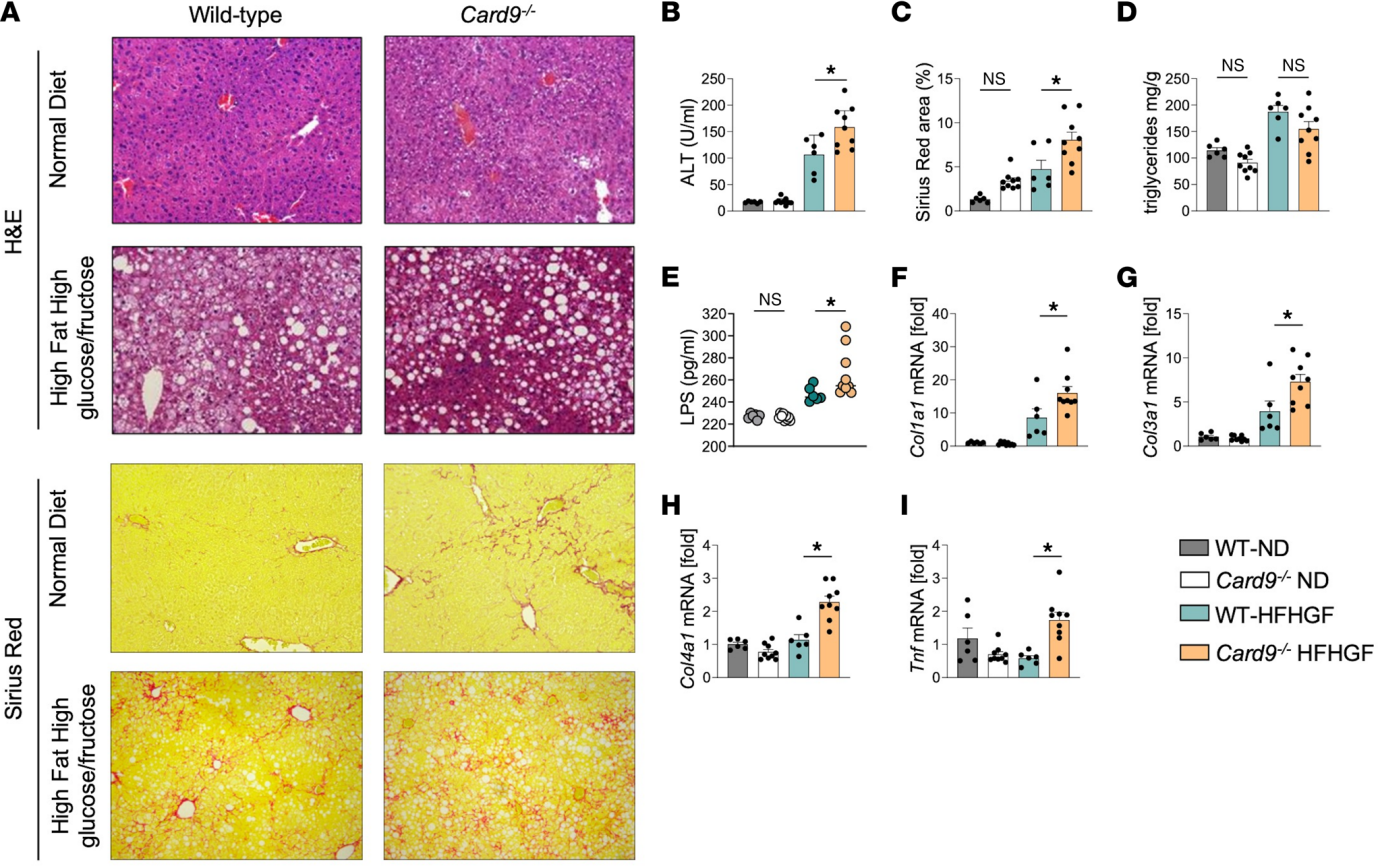
CARD9 deficiency exacerbates MASLD. (**A**) H&E and Sirius red staining of liver sections from WT mice fed normal diet (WT-ND, *n* = 6), CARD9-deficient mice fed ND (*Card9^–/–^* ND, *n* = 6), WT mice fed high-fat, high-glucose/-fructose diet (WT-HFHGF, *n* = 6), and *Card9^–/–^* mice fed HFHGF diet (*n* = 9). Original magnification, ×20. (**B**) Serum ALT. (**C**) Sirius red quantitation. (**D**) Hepatic triglyceride content. (**E**) Serum lipopolysaccharide (LPS). (**F**) *Col1a1* (**G**) *Col3a1* (**H**) *Col4a1* and (**I**) *Tnf* mRNA expression. Statistical significance was calculated with 1-way ANOVA followed by Holm-Šídák multiple-comparison test (**B**–**H**). *P < 0.05. NS, not significant.

Our human cohort and amphotericin B–treated mouse data suggested the importance of the gut mycobiota in MASLD fibrosis. We therefore focused on fungal immune system mediators. While MyD88 and NLRP3 have a broad range of effects, including responses to bacteria and fungi, CARD9 is a protein that has specific antifungal effects and has been well linked to the pathogenesis of inflammatory bowel disease and metabolic disease (21, 23). We subjected *Card9^–/–^* mice to a diet-induced steatotic liver disease to determine what role a lack of fungal immunity would have on the disease model progression of MASLD (24). After a 6-month period of HFHGF feeding, we found that *Card9^–/–^* mice exhibited worsened liver injury and fibrosis, but not worsened steatosis and hepatic triglyceride content when compared to their WT counterparts (Figure 4, A–D). Serum lipopolysaccharide (LPS) was also elevated under metabolic stress in *Card9^–/–^* mice (Figure 4E). These changes were accompanied by increased liver fibrosis markers, including *Col1a1*, *Col3a1*, and *Col4a1*. Furthermore, *Card9* deficiency increased the expression of proinflammatory marker *Tnf* (Figure 4, F–I). Notably, *Card9* deficiency by itself under ND conditions was not sufficient to lead to significant liver injury or fibrosis. These results demonstrate that CARD9 inhibits MASLD-induced liver injury, inflammation, and fibrosis, but not steatosis.

### Card9 deficiency in mice leads to microbial dysbiosis.

**Figure 5:**
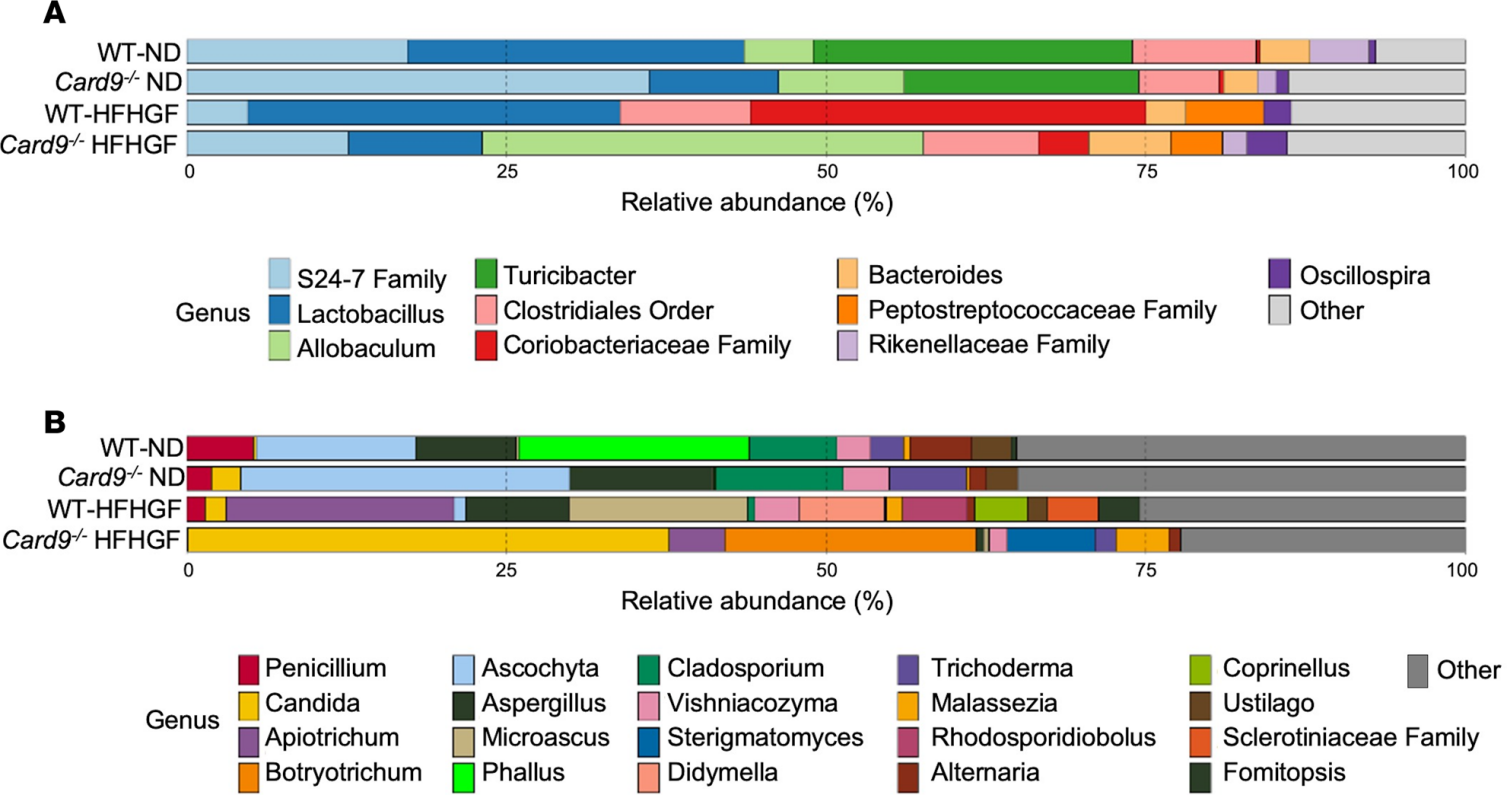
CARD9 deficiency causes microbial dysbiosis. WT mice fed normal diet (WT-ND, *n* = 6), CARD9-deficient mice fed ND (*Card9^–/–^* ND, *n* = 6), WT mice fed high-fat, high-glucose/-fructose diet (WT-HFHGF, *n* = 6), and *Card9^–/–^* mice fed HFHGF diet (*n* = 9). (**A**) Top 10 bacterial genera by relative abundance determined by *16S* sequencing of stool and (**B**) top 20 fungal genera determined by *ITS1* sequencing. Data represented as average relative abundance.

We next sought to determine what changes might have occurred at the microbiota level given CARD9’s role in fungal immunity. Large microbiota changes were apparent within both bacteria and fungi in the murine MASLD (Figure 5). *Card9^–/–^* mice appear to have slightly more *S24-7* family under normal and HFHGF diet feeding compared with controls, although it appears diminished under HFHGF feeding at baseline. *Allobaculum* showed a similar trend within *Card9^–/–^* mice; there was a large expansion of this genus under HFHGF feeding. *Lactobacillus*, on the other hand, showed the opposite trend; it was diminished at both baseline and under HFHGF in *Card9^–/–^* mice (Figure 5A). *Coriobacteriaaceae* and *Peptostreptococcaceae* families appear to have expanded in HFHGF feeding conditions but were less pronounced in the *Card9^–/–^* mice. In fungi, the largest differences occurred between ND-fed and HFHGF-fed animals, with minimal overlap in fungal species except for *Apiotrichum* (purple) (Figure 5B). Notably, the genera *Penicillium* (dark red) (Figure 5B) and *Ascochyta* (light blue) (Figure 5B) were abundant in the ND groups, *Ascochyta* in fact was more abundant in the *Card9^–/–^* as compared with the WT ND groups. Both genera showed substantial reductions in the WT HFHS group, with *Penicillium* being nearly undetectable in the *Card9^–/–^* mice on an HFHGF diet (Figure 5B). These results parallel the similar reduction in *Penicillium* we detected in our human cohort. Additionally, *Candida*, which was absent in WT animals under ND conditions, was detected at baseline in the *Card9^–/–^* mice and expanded under HFHGF feeding. Taken together, these results indicate that CARD9 deficiency has profound effects on inducing a gut microbial dysbiosis that only worsens under conditions of metabolic stress.

**Figure 6:**
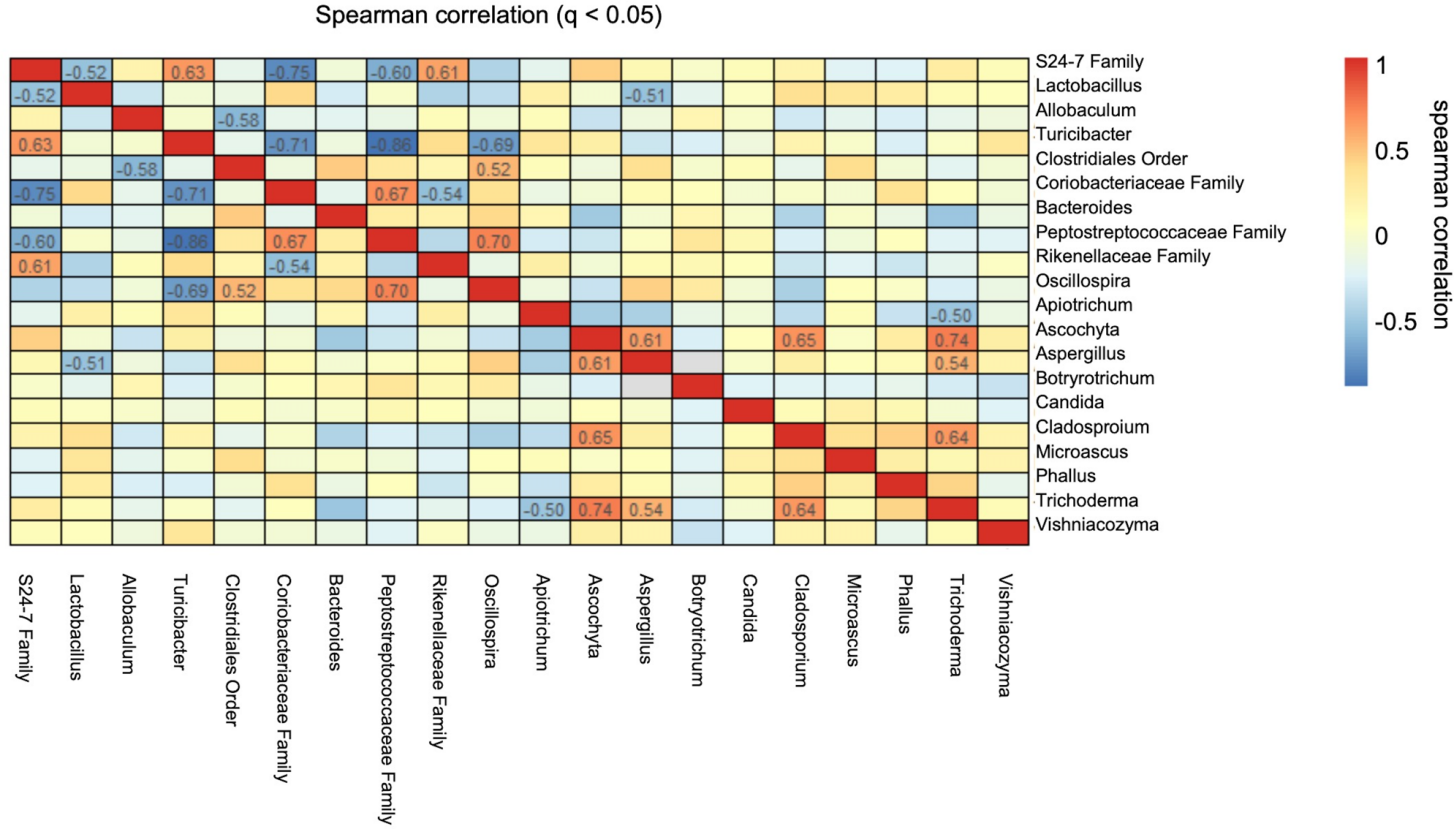
Spearman’s correlation between top 10 fungi and bacteria found in the *Card9^–/–^* animal cohort. Cells with numbers represent Spearman’s correlation coefficients of less than –0.3 or greater than 0.3 that were found to be significant.

We performed Spearman’s correlation analysis, comparing the top 10 bacteria to the top 10 fungi across all our data sets to determine whether the reduction in *Penicillium* was merely an epiphenomenon emerging from the overgrowth of other organisms (Figure 6). We found a significant inverse correlation between *Aspergillus* and *Lactobacillus*, a bacteria thought to be beneficial (25). In terms of correlations between fungi, significant positive correlations were found between the following pairs: (a) *Ascochyta*: *Trichoderma*, *Cladosporium*, *Aspergillus*; (b) *Aspergillus*: *Ascochyta*, *Trichoderma*; (c) *Cladosporium*: *Trichoderma* and (d) one inverse correlation with *Trichoderma* and *Apiotrichum*. Numerous significant correlations were found between bacteria. Notably, the *S24-7* family, a genus that consumes mucin, was found to be correlated with *Lactobacillus*, *Turicibacter*, *Bacteroides*, the *Peptostreptococcaceae* family, and the *Rikenellaceae* family (26). The *Peptostreptococcae* family, which has also been correlated with MASLD, was found to be inversely correlated with *Turicibacter*, the aforementioned *S24-7* family, and positively correlated with the *Coriobacteriaceae* family and *Oscillospira* (27).

### CARD9 expression extends beyond myeloid cells to include intestinal enterocytes.

*CARD9* expression has been well described to be expressed within in myeloid cells, so we sought to identify the tissue compartment driving liver injury in this model. We therefore examined *CARD9* expression in the small intestine and liver, the 2 main compartments where a disease phenotype was present (microbial dysbiosis and liver injury/fibrosis). To accomplish this, we used publicly available single-cell RNA sequencing (scRNA-seq) data sets that examined the murine small intestine under normal and high-fat feeding conditions and the human liver (28, 29).

**Figure 7:**
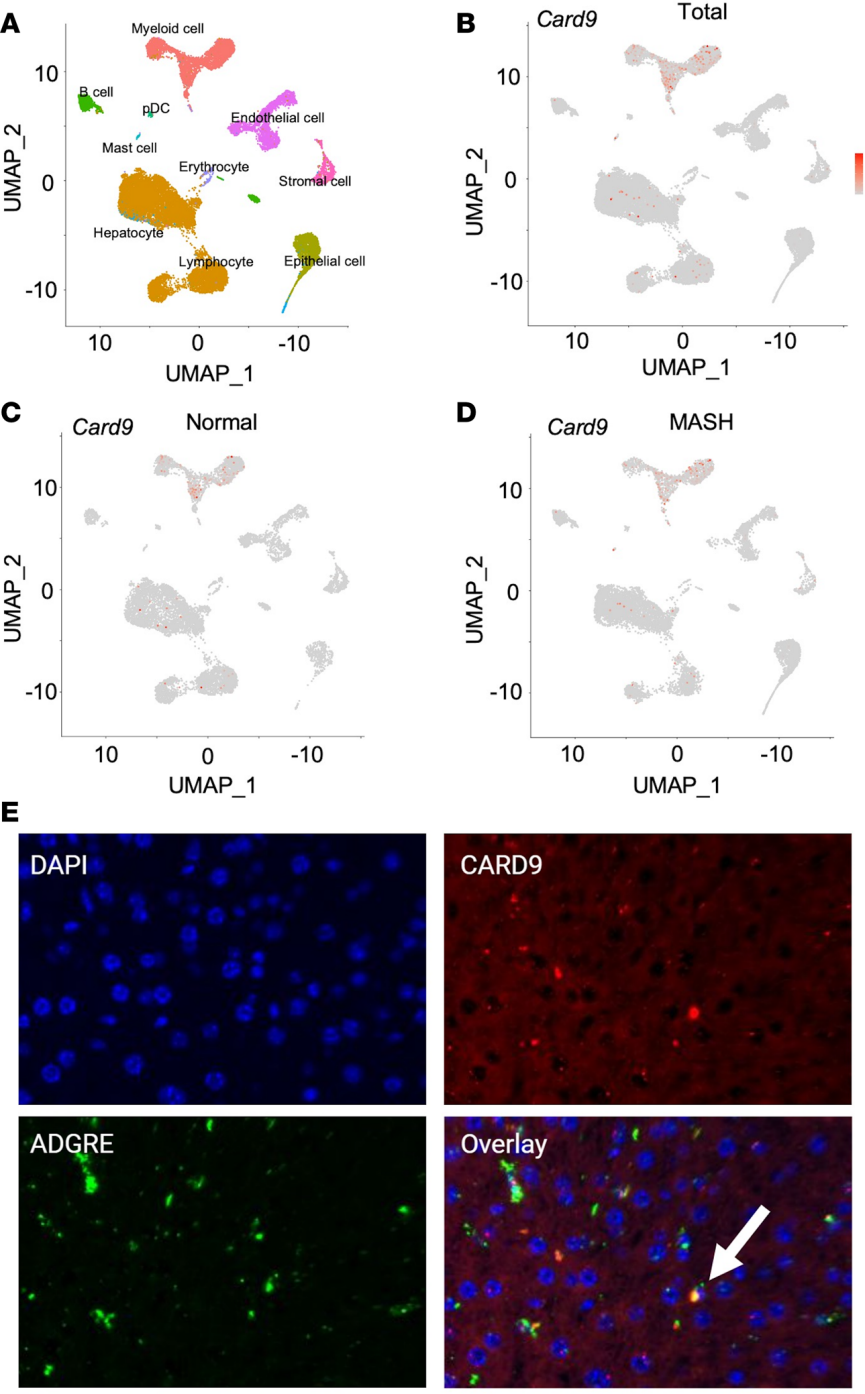
*CARD9* expression is found in a subset of human liver and murine macrophages. (**A**) scRNA-seq data analyzed from database SCP2154 showing expression of (**B**) *CARD9* in macrophages. *CARD9* expression patterns in both normal (**C**) and metabolic dysfunction–associated steatohepatitis (MASH) conditions (**D**) remain relatively unchanged in macrophages. (**E**) RNA in situ hybridization targeting *Adgre* (F4/80) and *Card9* in WT murine liver. Overlap of *Card9* and *Adgre* probe in nuclei indicate coexpression. Representative microscopic images of 3 independent experiments are shown. Original magnification, ×40.

We first investigated the *CARD9* expression in human liver. We found that *CARD9* is primarily expressed in liver myeloid cells, with some scattered expression within hepatocytes and lymphocytes as shown by scRNA-seq analysis (Figure 7, A and B), and with no changes in expression pattern between non-injured and MASH livers (Figure 7, C and D). We confirmed this finding in murine liver using RNA in situ hybridization targeting both the gene for F4/80, encoded by *Adgre*, a murine macrophage marker, and *Card9*, highlighting their coexpression in specific macrophage populations in the liver (Figure 7E). We found only limited expression of *CARD9* in hepatocytes.

**Figure 8:**
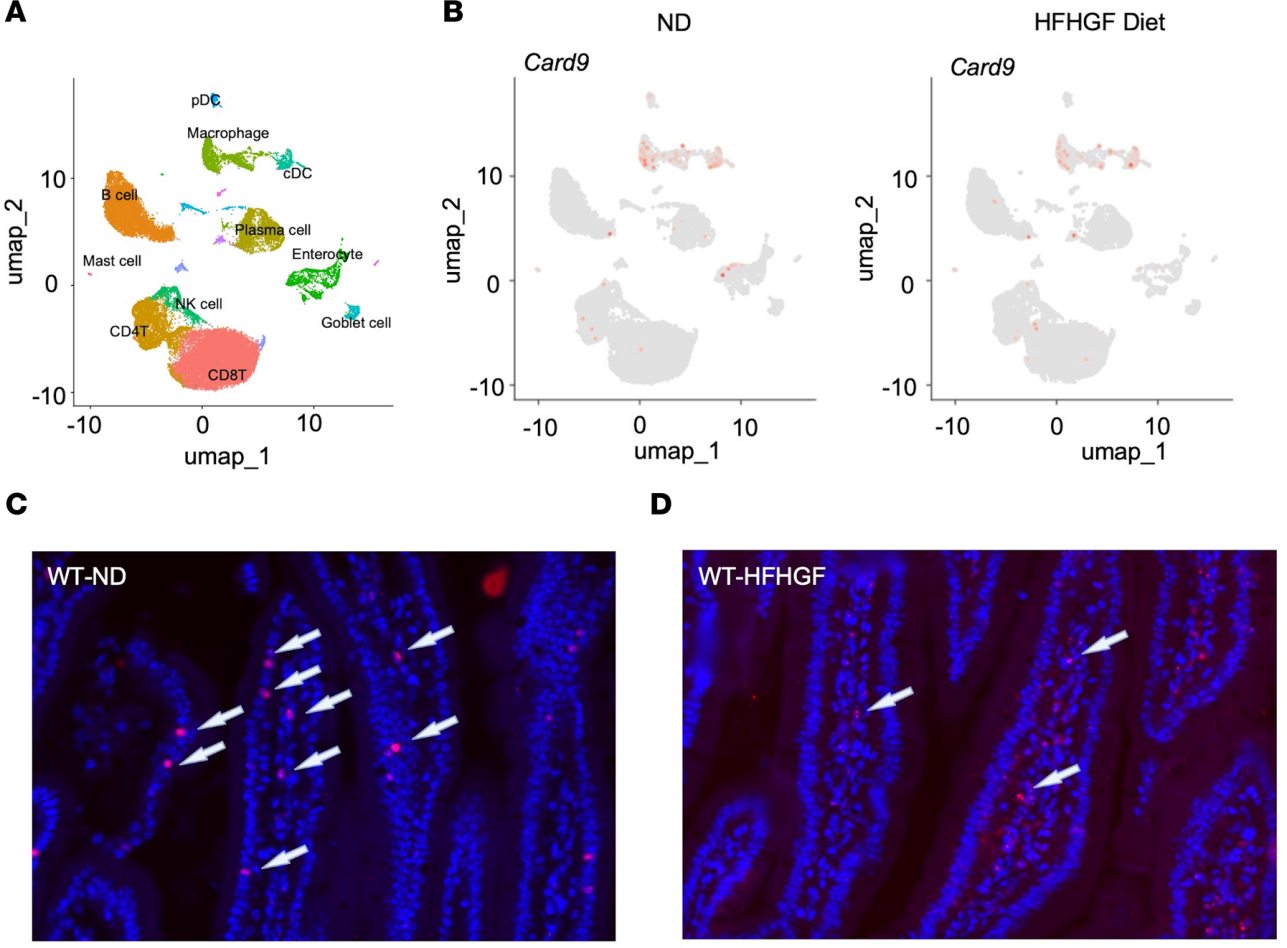
Card9 is expression is not restricted to myeloid cells. Analysis of scRNA-seq data from GSE221006 examining the expression of *Card9* in murine small intestine under normal diet (ND) and high-fat, high-glucose/-fructose (HFHGF) diet conditions (29). RNA in situ hybridization targeting *Card9* in murine small intestine. Expression was detected in both enterocytes and in the lamina propria (arrows) in ND (**C**) and was almost entirely absent in enterocytes under HFHGF diet conditions (**D**). Representative microscopic images of 3 independent experiments are shown. (**C** and **D**) Original magnification, ×40.

Since we did not observe differences in *CARD9* expression between normal and MASLD livers but did observe increased gut permeability in *Card9^–/–^* mice as evidenced by increased LPS levels, we analyzed *Card9* expression in the intestine as the mechanism of increased gut permeability in MASLD. We confirmed that *Card9* is expressed mostly in intestinal murine macrophages, as reported with some scattered expression in T, B, and plasma and dendritic cells (Figure 8A) (30). Notably, *Card9* was also present in enterocytes. Under HFHGF diet feeding, *Card9* expression was similar in intestinal myeloid cells but diminished in enterocytes (Figure 8B). Given the low overall low quality of the data, we sought to confirm whether this finding was true in mouse small intestine. We therefore utilized RNA in situ hybridization to directly examine *Card9* expression within the murine small intestine. We confirmed robust *Card9* expression in enterocytes under ND conditions (Figure 8C) but a marked reduction under HFHGF diet feeding (Figure 8D). Of note, the cells expressing *Card9* did not appear to be expressed in crypts where Paneth cells reside. Cells expressing *Card9* within the lamina propria are expected to be mostly macrophage in origin given the scRNA-seq data. This expression data along with the observed increased intestinal permeability suggest the pivotal role of CARD9 in enterocytes for MASLD development.

### Functional role of CARD9 in the intestinal epithelium.

**Figure 9:**
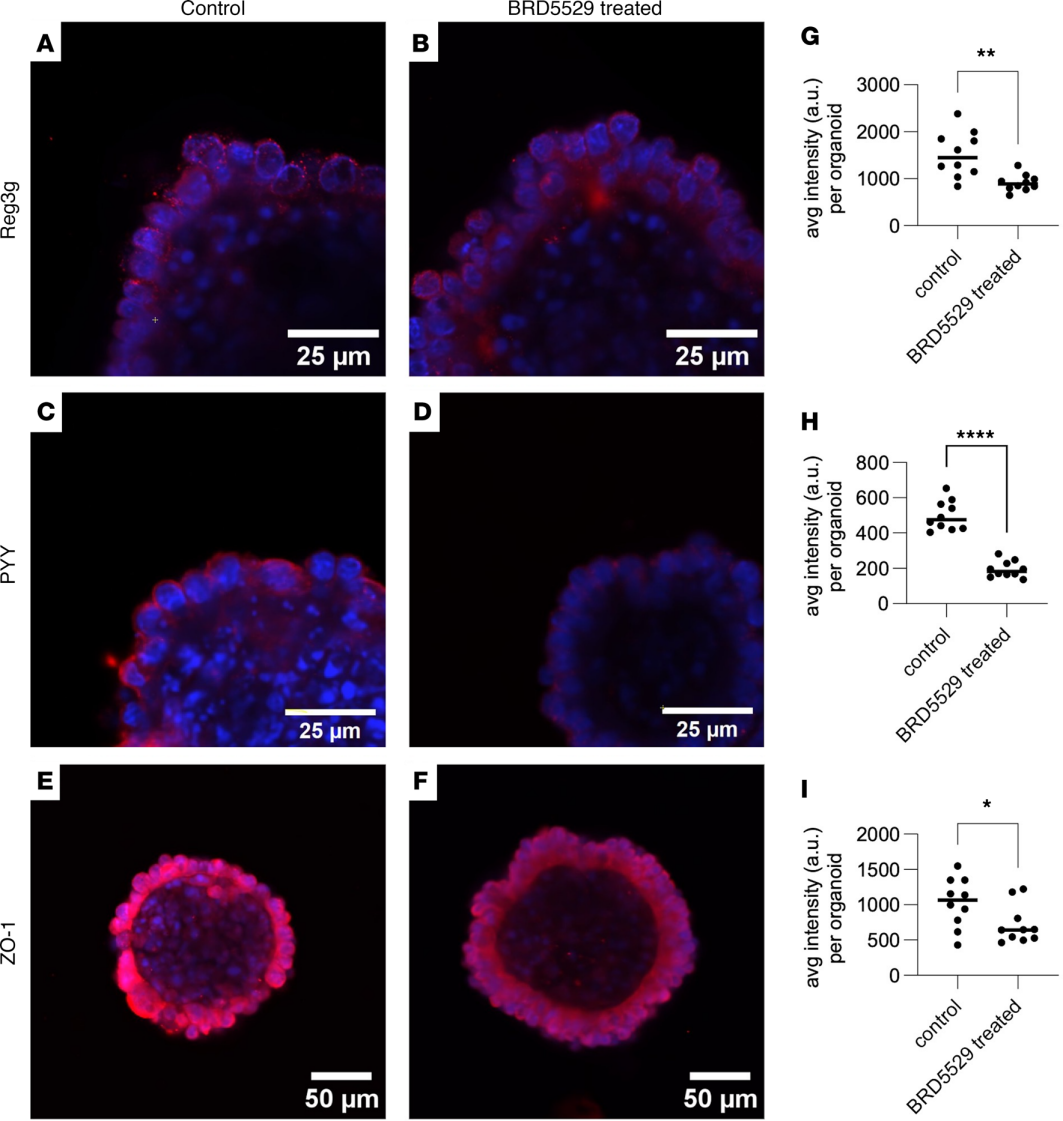
CARD9 inhibition leads to reduced expression of Reg3g in WT small intestinal organoids. (**A**, **C**, and **E**) Whole-cell mount of WT small intestinal organoids showing positive immunofluorescence in small granules for Reg3g (**A**), peptide YY (PYY, **C**), and ZO-1 (**E**) in enterocytes counterstained with DAPI (blue). (**B**, **D**, and **F**) Exposure of organoids to 2 μM BRD5529, a CARD9-specific inhibitor, results in a marked decrease in expression of Reg3g (**B**), PYY (**D**), and ZO-1 (**F**). Representative microscopic images of 3 independent experiments are shown. Average fluorescence intensity per organoid for (**G**) Reg3g, (**H**) PYY, (**I**) ZO-1. *n* = 10 randomly selected organoids. Statistical significance was calculated with 2-tailed Student’s *t* test (**G**–**I**). *P < 0.05, **P < 0.01, ****P < 0.0001.

Given the finding that *CARD9* was found expressed in enterocytes, we sought to understand whether there was any functional consequence to its expression in enterocytes. Prior literature indicated that *Card9^–/–^* mice had a reduction in Reg3g, an antimicrobial peptide that could be used to regulate the gut microbiota and is notably expressed in enterocytes and not immune cells (31). We therefore derived intestinal organoids, which recapitulate all enterocyte lineages, from WT mice (32). Using this system, we examined the expression of Reg3g, peptide YY (PYY), an antifungal peptide, and ZO-1, a tight junction molecule, in the small intestine. We found robust expression of Reg3g, PYY, and ZO-1 in cells as evidenced by positive immunofluorescence in the epithelial enterocytes (Figure 9, A, C, and E). We then exposed intestinal organoids to BRD55229, a CARD9-specific inhibitor (33). Under CARD9-inhibited conditions, we found Reg3g, PYY, and ZO-1 expression to all be markedly diminished (Figure 9, B, D, and F–I). These results indicate that under steady-state conditions, CARD9 function is required to help drive the expression of these critical gut barrier function proteins, which then regulate gut bacterial and fungal composition and gut permeability for MASLD development.

## Discussion

The aim of this study was to explore the role of gut fungi in MASLD progression. Our study began by examining the composition of gut fungi in a diverse cohort of patients with MASLD with both mild and severe fibrosis. We found that patients with more advanced fibrosis harbored a different composition of fungi. Specifically, the genus *Penicillium* was diminished within this population. We sought to examine fungal dysbiosis in an animal model and chose *Card9* deficiency given its specificity for fungal recognition in myeloid cells. Under the context of prolonged metabolic stress, *Card9^–/–^* mice exhibited worsened liver injury and fibrosis. When we examined their gut mycobiota, fungal dysbiosis was also apparent and like our human cohort also had a diminished abundance of the *Penicillium* genus. These findings indicate that fungal dysbiosis is linked to MASLD progression but also that *Penicillium* may be a beneficial fungus found within the gastrointestinal tract that may help to ameliorate or prevent disease progression. We next turned our attention to understanding where *Card9* was specifically expressed and found that it was primarily expressed in liver and intestinal macrophages of both mice and humans. We found that *Card9* expression is also expressed in intestinal enterocytes and this appeared to be diminished under HFHGF diet feeding conditions in mice. We confirmed these results in mouse small intestines using RNA in situ hybridization. Given the finding of *Card9* expression in the intestine, we next sought to understand the functional consequences of *CARD9* in this intestinal compartment. We therefore derived intestinal organoids and found that under *CARD9* inhibition, organoids exhibited less expression of antibacterial Reg3g, antifungal PYY, and tight junction molecule ZO-1. Together, these results indicate that CARD9 helps to maintain the epithelial barrier and modulate both gut bacteria and fungi, which together help prevent liver injury and fibrosis through diminished translocation of gut microbial products, such as LPS.

While most previous studies have focused on the role of gut bacteria, our findings support emerging evidence that fungal dysbiosis may also contribute to MASLD disease progression. The observation that *Penicillium* is diminished in both patients with MASLD with advanced liver fibrosis and *Card9^–/–^* mice suggests a potential beneficial role for this fungus in maintaining homeostasis within the gut-liver axis. The potential for *Penicillium*-derived metabolites to exert direct effects on liver health warrants further consideration. These metabolites may modulate hepatic inflammation, oxidative stress or fibrotic pathways and offer protection against liver injury. For example, mycophenolic acid, produced by some strains of *Penicillium*, is used as an immunosuppressant for liver transplant patients (34). This hypothesis aligns with the notion that microbial metabolites can have systemic effects beyond the gut.

This association between *Penicillium* and improved liver outcomes could also reflect its role in maintaining gut microbial homeostasis. *Penicillium* species are known to produce metabolites with antifungal properties, such as peptides that inhibit pathogenic fungi like *Candida* (35, 36). Additionally, *Penicillium*’s historical significance as a source of penicillin highlights its well-established antibacterial effects, which further underscores its role in shaping microbial ecosystems by limiting the production of harmful bacteria (37). This potential dual role — as a regulator of microbial homeostasis and a potential producer of beneficial metabolites — positions *Penicillium* as a potential pivotal genus in maintaining gut-liver axis health. Future studies will be needed to identify and characterize these specific metabolites and their role in regulating the microbiome and their effects on liver health. Leveraging these effects may be useful in developing therapeutic drugs that might mitigate liver disease progression.

We cannot entirely exclude the possibility that the observed reduction in the *Penicillium* genus reflects competitive displacement by other microbes occupying a similar ecological niche, rather than a direct functional role. For instance, *Aspergillus* was inversely correlated with *Lactobacillus* in our dataset, a genus widely recognized for its beneficial properties (25). Both *Naganishia* and *Aspergillus* were also detected in animals treated with amphotericin B. Notably, *Aspergillus* is a well-documented pathogenic environmental fungus, while the *Naganishia* genus, formerly classified under *Cryptococcus*, includes species commonly associated with infections in immunocompromised hosts and is frequently found in the environment (38, 39). These observations support the hypothesis that the overgrowth of potentially pathogenic fungi may suppress other fungal taxa, some of which may confer benefits to the host.

Interestingly, several fungal taxa displayed significant correlations with specific bacterial genera, suggesting potential cross-kingdom interactions that may influence microbial community structure and host outcomes. For example, the negative correlation between *Aspergillus* and *Lactobacillus* could reflect competitive exclusion or antagonism. Conversely, some fungi may be positively correlated with commensal or beneficial bacteria, raising the possibility of synergistic interactions that promote host health. Deciphering these relationships will be crucial to understanding whether fungal dynamics act independently or in concert with bacterial populations in shaping the gut environment. Further work is needed to determine whether *Penicillium* plays such a role in this process and could be addressed in the future through targeted gavage experiments using specific *Penicillium* species, combined with longitudinal microbiome profiling to assess downstream effects on the microbiome and the resultant liver phenotype.

Interestingly, CARD9, which has been extensively studied for its role in fungal recognition and immune signaling within myeloid cells, was also found to be expressed in intestinal enterocytes. This intriguing finding suggests an additional role for CARD9 particularly due to its localization at the critical interface between the gut lumen and the gut microbiota. This finding expands the functional repertoire of CARD9 beyond immune modulation to include epithelial homeostasis and gut barrier maintenance. The diminished expression of antimicrobial peptide Reg3g and the tight junction protein ZO-1 in CARD9-inhibited organoids provides evidence that CARD9 is essential in promoting gut barrier function. This reduction in Reg3g mirrors findings seen in other models where CARD9 knockouts were used (21). Additional compounding effects of loss of ZO-1 and PYY may also contribute to disease progression (40). Because antifungal PPY, which is regulated by CARD9, inhibits *Candida* growth, the loss of antifungal PYY by CARD9 inhibition may support the growth of MASLD-promoting fungi, including *Candida* (40). Conversely, dominance of *Candida* may suppress the growth of *Penicillium*, which further enhances *Candida* growth in a feed-forward mechanism. These complex mechanisms governed by CARD9 in the intestines likely facilitate microbial translocation, which triggers inflammatory responses in the liver.

Interestingly, *Card9* deficiency by itself was not able to recapitulate the liver injury phenotype. Liver disease only manifested when *Card9^–/–^* animals were subjected to metabolic stress. This observation suggests that an additional “hit” likely provided by the metabolic stress is necessary to drive disease progression. These findings align with the “multiple-hit” hypothesis of liver disease where a combination of factors coincide to exacerbate tissue injury. Under these conditions, we found that *Card9* expression appeared to be diminished, particularly in intestinal enterocytes. Enterocytes, which are directly exposed to dietary fatty acids and other metabolites may experience their own version of metabolic stress when under a high nutrient load which may lead to metabolic and transcriptional changes that suppress *Card9* expression. Future studies are needed to explore the mechanisms by which metabolic stress modulates CARD9 expression in enterocytes. A key area of focus would include determining whether restoring CARD9 expression under high-fat feeding conditions could ameliorate disease progression.

This study highlights the critical role of CARD9 in maintaining gut barrier integrity under metabolic stress and its contribution to regulating fungal dysbiosis and gut-liver axis health. These results build on emerging evidence that fungal dysbiosis can contribute to liver disease progression and may have the potential to identify potential targets within the gut microbiome itself. Understanding CARD9’s dual role in microbial homeostasis and gut barrier integrity could pave the way for novel MASLD therapies. Future studies should explore the mechanisms of CARD9-mediated fungal immunity in patients with MASLD. Overall, our findings highlight the importance of the gut-liver axis in MASLD and provide insights into the interplay between gut fungi, microbial dysbiosis, and liver health.

## Methods

### Sex as a biological variable.

Both male and female patients were included in our human cohort study of MASLD. Animal studies only included male mice to maximize the disease phenotype. MASLD and MASH have been found to be more prevalent in men, according to population studies (41).

### Next-generation sequencing of stool DNA.

Stool samples from humans or mice were collected and stored at –80°C until processed. DNA was extracted using the QIAamp PowerFecal Pro DNA Kit (Qiagen) using the modified directions for tough-to-process samples. Purified DNA was then quantitated on a Nanodrop (Thermo Fisher Scientific). DNA was provided to the Cedars Sinai Medical Center Genomics Core for sequencing amplifying either the bacterial *16S* ribosomal DNA (rDNA) (8F: 5′-AGAGTTTGATCMTGGCTCAG-3′; 357R: 5′-CTGCTGCCTYCCGTA-3′) or the fungal *ITS1* (ITS1f: 5′-CTTGGTCATTTAGAGGAAGTAA-3′; ITS2: 5′-GCTGCGTTCTTCATCGATGC-3′). Raw data were provided in FASTQ format as 2 × 300 paired-end sequences. QIIME2 was used to demultiplex data and prepare data for further downstream processing (42). *16S* feature tables were generated after denoising data using the DADA2 plugin available in QIIME. Taxonomy was assigned using the GreenGenes 99% OTU database (43). ITS1 data were first preprocessed using ITSxpress plugin to trim the internally transcribed spacer sequence prior to processing (44). Fungal sequences were aligned to the UNITEv9 dynamic database (45). QZA objects were then exported into the Rstudio environment (version 2024.09.0) using the qiime2R package (https://github.com/jbisanz/qiime2R). The phyloseq package was used for further data processing and the microviz package was used for data visualization (46, 47).

### Random forest classification.

Random forest classification was done using the caret package (https://github.com/topepo/caret/) in the R programming environment. Briefly *16S* and *ITS1* relative abundance tables were combined and any feature columns missing greater than 30% were removed. The number of trees used in the final model was 500 and the variable importance feature was used to extract the most important features in the data set.

### Animals.

C57BL/6J mice were purchased from The Jackson Laboratory and housed in groups of 3–4 mice. *Card9^–/–^* mice were provided by David Underhill at Cedars Sinai Medical Center. At 8 weeks of age, mice were provided ad libitum access to either an ND or a high-fat diet (Research Diets Inc. D12492, 60% kcal fat) supplemented with fructose (23.1 g/L) and glucose (18.9 g/L) for 6 months. Amphotericin B was dissolved in the drinking water at a concentration of 0.3 mg/mL. At the end of the study period, mice were euthanized and liver, intestines, and blood were collected.

### Histological analysis.

Formalin-fixed, paraffin embedded (FFPE) liver or intestinal tissue was used. H&E staining was used to assess basic architecture and Sirius red for detection of fibrosis. Images were processed using the Fiji image editing software (48).

### Serum ALT and AST.

Serum alanine aminotransferase (ALT) and AST levels were measured using the ALT or AST infinity reagent from Thermo Fisher Scientific, as described by the manufacturer’s protocol.

### Hepatic triglycerides.

Hepatic triglycerides were measured by first extracting triglycerides from hepatic liver tissue. Briefly, 100–300 mg of liver tissue was saponified using ethanolic KOH and incubated overnight at 55°C. Tissue was then vortexed to ensure complete digestion. The volume was then adjusted to 1.2 mL using 50% ethanol, spun, and 1 M MgCl_2_ was added to the mixture. The supernatant was then removed and triglycerides measured using the Triglyceride Liquid Reagent set (Pointe Scientific, Inc), as per the manufacturer’s instructions.

### LPS measurement.

Serum LPS was measured using the mouse LPS ELISA kit (Cusabio) per the manufacturer’s instructions.

### PCR analysis.

RNA was extracted from flash-frozen liver tissue using TRIzol (Life Technologies). Extracted RNA was reverse transcribed into DNA using iScript reverse transcription supermix from Bio-Rad. PCR was performed using SYBR Green. Quantification was performed after normalization to *18S* RNA levels. The following primers were used: *18S* (F: 5′-CGCTGAGCCAGTCAGTGT-3′; R: 5′-TAGAGGGACAAGTGGCGTTC-3′), *Col1a1* (F: 5′-TAGGCCATTGTGTATGCAGC-3′; R: 5′-ACATGTTCAGCTTTGTGGACC-3′), *Col3a1* (F: 5′-TAGGACTGACCAAGGTGGCT-3′), *Col4a1* (F: 5′-GGTGCTTCACAAACCGCACA-3′; R: 5′-CTTCGCCTCCAGGAACGACT-3′), and *Tnf* (F: 5′-CCACCACGCTCTTCTGTCTAC-3′; R: 5′-AGGGTCTGGGCCATAGAACT-3′).

### scRNA-seq data acquisition, processing, and analysis.

scRNA-seq datasets were obtained from publicly available repositories. GSE221006 data were downloaded from the NCBI Gene Expression Omnibus database (https://www.ncbi.nlm.nih.gov/geo/query/acc.cgi?acc=GSE221006). The dataset includes transcriptomics profiles of small intestinal cells from mice that were fed by ND and high-fat high-sucrose (HFHS) diets. SCP2154 data were accessed from the Single Cell Portal (https://singlecell.broadinstitute.org/single_cell/study/SCP2154), which contains integrated scRNA-seq data from 5 different human liver fibrosis studies (GSE185477, GSE125188, GSE156625, GSE192740, GSE115469, and GSE136103).

Processed count matrices were downloaded as provided by the repositories. GSE2210006 data were processed using the Cell Ranger pipeline (v 6.0, 10X Genomics) by the original authors. The count matrices were normalized and analyzed using the Seurat package (v5.1.0) through R. SCP2154 data were collected as preprocessed matrices and reanalyzed for consistency using the standard Seurat workflow.

Gene expression matrices were log-normalized, and variable genes were identified using the variance-stabilizing transformation. Principal component analysis (PCA) was performed to reduce dimensionality. Clustered data were visualized by uniform manifold approximation and projection (UMAP). Cell types were identified and annotated based on provided metadata in the original datasets. SCP2154 data were batch corrected by harmony packages. Feature plots of the *Card9* gene were generated by Seurat function.

### RNAscope.

RNAscope was performed to visualize expression of mRNA transcripts in FFPE liver or intestine using specific probes for *Card9* or *Adgre*. The RNAscope Multiplex Fluorescent V2 assay protocol and kit were used as per the manufacturer’s instructions. Briefly, FFPE sections were deparaffinized and pretreated per the manufacturer’s instructions for each tissue type. This was followed by multiple hybridization steps at 40°C. Slides were finally counterstained with DAPI.

### Intestinal organoids and immunofluorescence.

Intestinal organoids were derived from WT C57BL/6J mice. Briefly, mice were euthanized and the small intestine isolated. The small intestine was cleaned of fecal contents, washed in cold PBS, and cut into small pieces before being immersed in a dissociation buffer of PBS with 2.5 mM EDTA. Tissue was then left to shake for 30 minutes at 4°C. The dissociation buffer was then removed and tissue placed back into cold PBS. Intestinal crypts were collected by filtering the solution with a 70 μm cell strainer. FBS (10% final) was added to the solution to prevent autodigestion of collected crypts. Crypts were then spun down at 300*g* for 5 minutes at 4°C. The supernatant was removed and replaced with IntestiCult intestinal mouse organoid growth media (STEMCELL Technologies) containing Pen-Strep antibiotics (100 U/mL) and a Y-27632, a ROCK inhibitor (BD Biosciences, 562822). Crypts were then resuspended and added to growth factor–free Matrigel (Corning) at a ratio of 2:3 and plated in a 24-well culture plate using 50-μL aliquots. After gel hardening, the droplet was immersed in 500 μL of medium, which was replaced at least 3 times per week. For specific CARD9 inhibition, the compound BRD5529 was purchased from MedChemExpress (33). Organoids were exposed to 2 μM of this compound directly in organoid growth media for a 24-hour time period. Organoids were then harvested from Matrigel and stained for immunofluorescence using a previously described protocol (49). The following primary antibodies were used: ZO-1 (1:100; 40-2200, Thermo Fisher Scientific), Reg3g (1:100; PA5-50450, Thermo Fisher Scientific), and PYY (1:100; ab22663, Abcam). Alexa Fluor 568–conjugated goat anti-rabbit antibody was used as the secondary antibody at a dilution of 1:500. Fluorescence intensity per organoid was measured using Fiji by tracing individual organoids and using the measure command.

### Statistics.

All statistical analyses were performed with a 1-way ANOVA followed by Holm-Šídák multiple-comparison test or by standard unpaired *t* test or χ^2^ test. A *P* value of less than 0.05 was considered significant.

### Study approval.

Samples from patients with MASLD were analyzed. The study was approved by the Cedars-Sinai Medical Center Institutional Review Board (IRB no. 42709). Written informed consent was obtained from all participants. All animal experiments were approved by the Cedars-Sinai Medical Center Institutional Animal Care and Use Committee (IACUC no. 8412).

### Data availability.

Raw sequencing data (*16S* and *ITS1*) are available in the NCBI Sequence Read Archive under accession no. PRJNA1203248. Data used to generate each figure are provided in the Supporting Data Values file.

## Author contributions

VP and ES designed the research studies. VP, SYK, TT, SHB, SL, and JK conducted experiments. YVP, SYK, TT, SHB, and JK acquired data. VP, SYK, TT, SHB, JK, and ES analyzed data. VP and ES wrote the original manuscript draft, which was reviewed and edited by VP, DMU, MN, SCL, and ES. VP and ES acquired funding. VP, DMU, MN, SCL, and ES provided resources. ES supervised the study.

## Conflict of interest

The authors have declared that no conflict of interest exists.

## Funding support

This work is the result of NIH funding, in whole or in part, and is subject to the NIH Public Access Policy. Through acceptance of this federal funding, the NIH has been given a right to make the work publicly available in PubMed Central.

- NIH grants R01DK085252 and R01DK138591 (to ES).
- Cedars-Sinai Cancer Community Outreach and Engagement Development Fund (to ES and VP).
- Clinical Scholar Award from Cedars-Sinai Medical Center (to VP).
- American Liver Foundation Liver Scholar Award (to VP).
- Pilot and Feasibility Grant from NIH P50AA011999 (to VP).

## Supplementary Material

Supplemental data

Supporting data values

## Figures and Tables

**Table 1:**
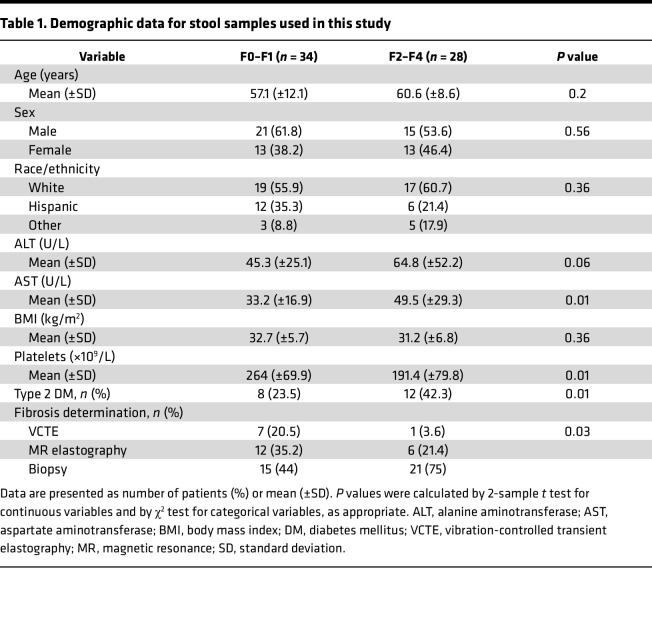
Demographic data for stool samples used in this study

## Version 1 03/10/2026

In-Press Preview

## Version 2 04/22/2026

Electronic publication
