# Supplementary material for: Loss of fungal sensing exacerbates liver injury in a murine model of MASLD
Source: JCI Insight. 2026 Mar 10;11(8):e190690. doi: 10.1172/jci.insight.190690 (PMC13135407; doi:10.1172/jci.insight.190690)
Supplement: Supplemental data [file jciinsight-11-190690-s015.pdf]

Supplemental Materials

Loss of fungal sensing exacerbates liver injury in a murine model of MASLD

Vijay Pandyarajan[1], So Yeon Kim[1], Mazen Noureddin[1,3,4], Takashi Tsuchiya[1], Selena Liu[1], Sadam Bhat[1], Jieun Kim[1], David M. Underhill[1,2], Shelly C. Lu[1], and Ekihiro Seki[1]

[1]Karsh Division of Gastroenterology and Hepatology, Department of Medicine, Cedars-Sinai Medical Center, Los Angeles, CA 90048, USA. [2]Department of Biomedical Sciences, Cedars-Sinai Medical Center, Los Angeles, CA 90048, USA. [3]Houston Methodist Hospital, Houston, TX 77030, USA. [4]Houston Research Institute, Houston, TX 77030, USA.

Corresponding author:

Ekihiro Seki MD, PhD,

Karsh Division of Gastroenterology and Hepatology, Department of Medicine, Cedars-Sinai Medical Center

8700 Beverly Blvd, Davis Building 2099, Los Angeles, CA 90048

Phone: +1-310-423-6605

Fax: +1-310-423-0157

E-mail: Ekihiro.Seki@cshs.org

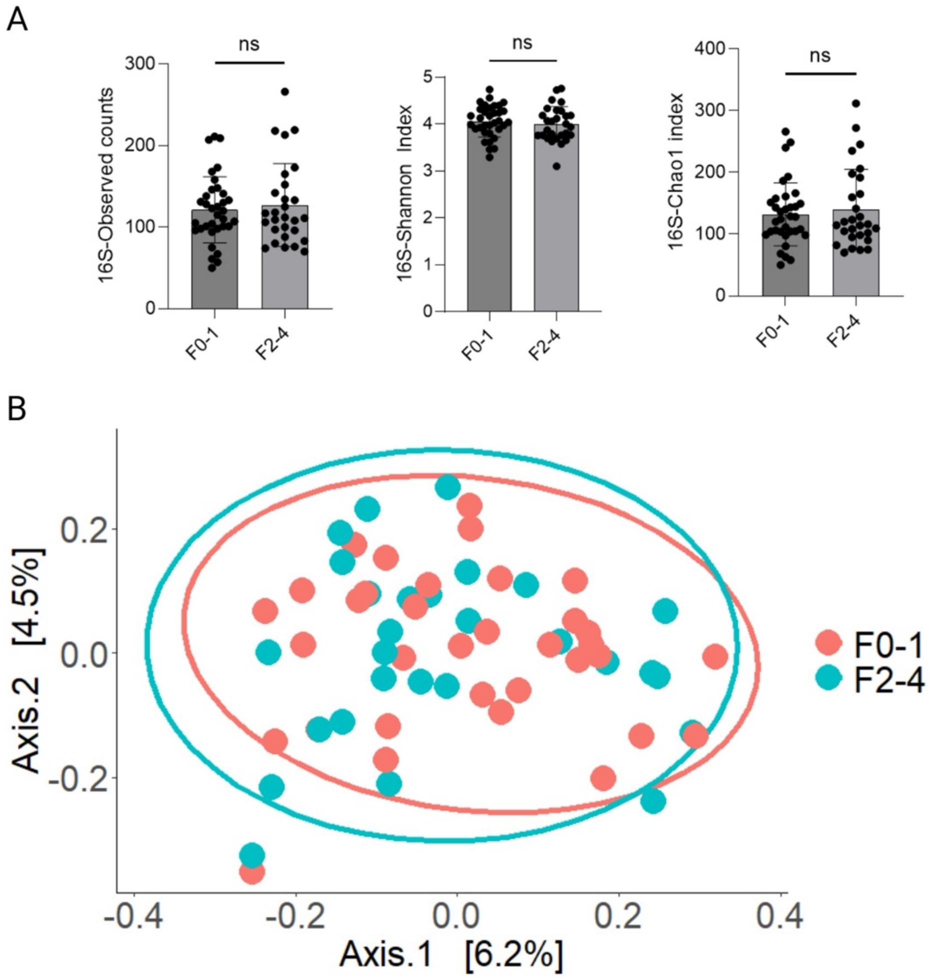

Supplementary Figure 1. 16S alpha and beta diversity. (A) Observed, Shannon and Chao1 alpha diversity indices were calculated for both groups in our human cohort. Data plotted as individual points and bars representing the mean +/- standard deviation. There were no significant differences as measured by unpaired t-tests ($p > 0.05$). ns, not significant. (B) Beta diversity plot showing overlapping centroids and was found to be non-significant by PERMANOVA analysis ($p > 0.05$).

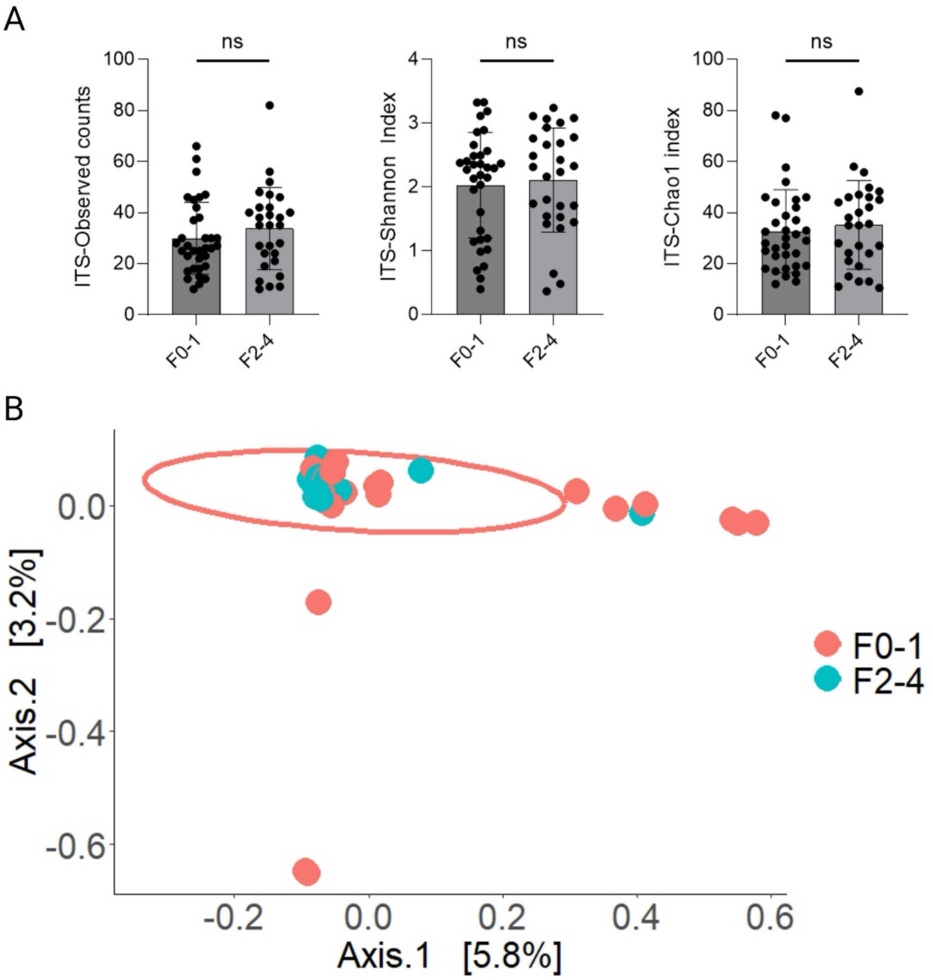

Supplementary Figure 2. ITS alpha and beta diversity. (A) Observed, Shannon and Chao1 alpha diversity indices were calculated for both groups in our human cohort. Data plotted as individual points and bars representing the mean +/- standard deviation. There were no significant differences as measured by unpaired t-tests ($p > 0.05$). ns, not significant. (B) Beta diversity plot of ITS data. These groups were found to be significantly different by PERMANOVA analysis ($p < 0.05$).
